# Learned young: HIV-1 germline-targeting vaccination in infants

**DOI:** 10.1097/COH.0000000000001051

**Published:** 2026-07-06

**Authors:** Rogier W. Sanders, Tom G. Caniels, John P. Moore, Sallie R. Permar

**Affiliations:** a Department of Microbiology and Immunology, Weill Cornell Medicine, New York, New York, USA; b Department of Medical Microbiology and Infection Prevention, Amsterdam University Medical Center; c Amsterdam Institute for Global Health and Development, Amsterdam, Netherlands; d Department of Pediatrics, Weill Cornell Medicine, New York, New York, USA

**Keywords:** broadly neutralizing antibodies, germline-targeting vaccine, HIV-1, pediatric vaccine

## Abstract

**Purpose of review:**

It is standard practice to protect infants against viruses that they are exposed to early in life, during childhood, or as adults. While HIV-1 is only rarely a risk in the period between the end of breast-feeding and the onset of sexual maturity, there are good immunological and practical arguments for assessing whether vaccination of young children might be beneficial.

**Recent findings:**

Studies of HIV-1-infected individuals suggest that the characteristics of the developing immune system may favor the evolution of B cells that produce broadly neutralizing antibodies (bNAbs), when compared to the same processes in adults. One way to exploit these properties of the infant immune system is targeting the germline precursors of B cells that can, over time and with the right sequential antigenic stimuli, mature into bNAb-producing plasma cells. Multidose immunization over time is standard practice within global childhood vaccine frameworks.

**Summary:**

We discuss strategies to accomplish the goal of inducing bNAbs, based on the use of specifically designed envelope glycoprotein immunogens. We also summarize how bNAbs may be used for passive protection of infants and combined with active vaccination.

## INTRODUCTION

The HIV-1 pandemic remains a major health burden worldwide. In 2024, 40.8 million people were living with HIV-1, there were 1.3 million new infections and 630 000 deaths from AIDS-related illnesses occurred (www.unaids.org). Sub-Saharan Africa remains the most affected region. While infection rates have gradually declined over the last two decades, disruption of the PEPFAR and USAID programs as a result of policy changes in the USA seem likely to compromise this progress (www.impactcounter.com). Young women and infants are among the most affected by the pandemic: globally, 1.4 million children were living with HIV-1 in 2024 with 120 000 new infant infections in that year (www.unaids.org). 

**Box 1:**
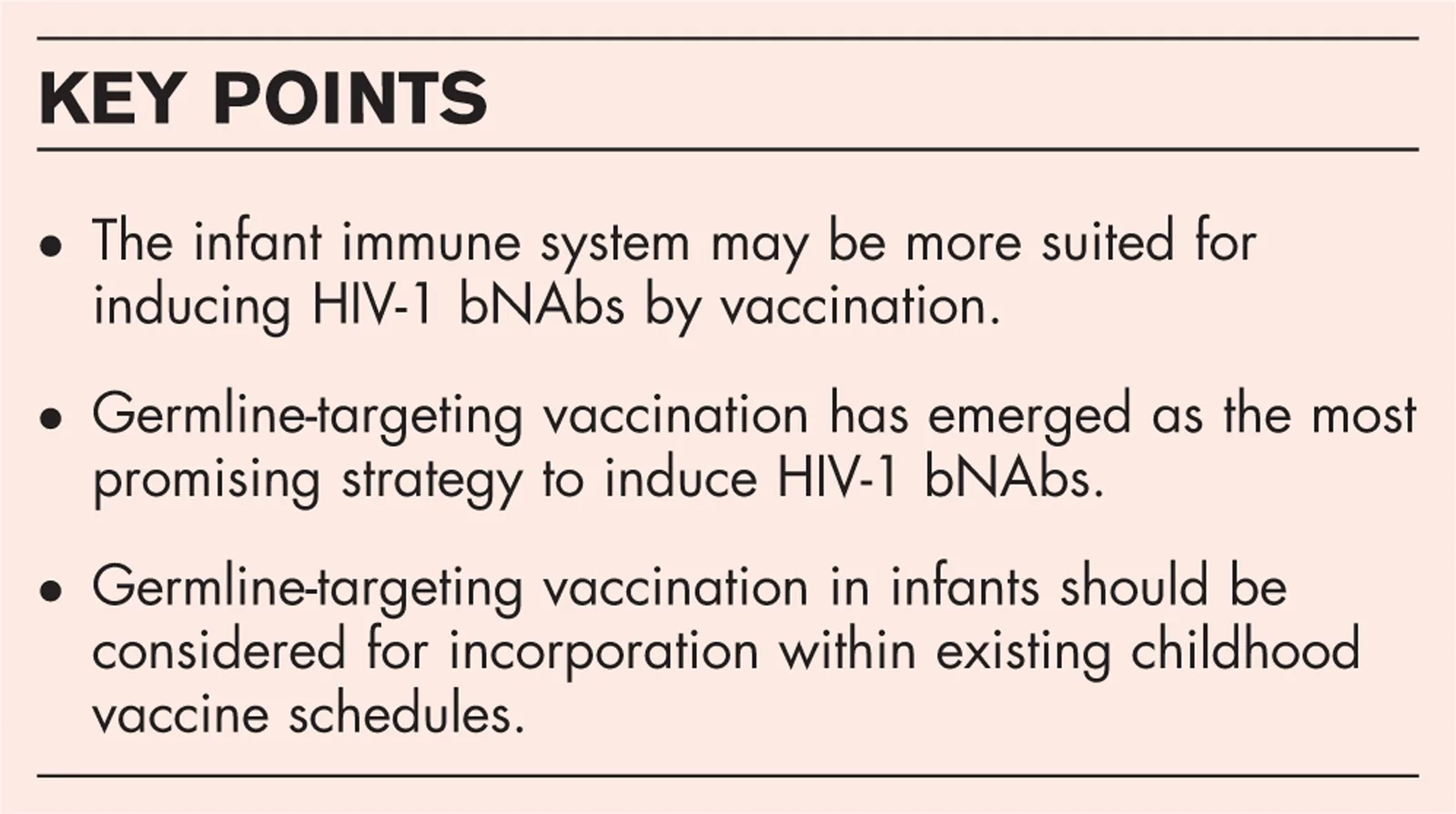
no caption available

**FIGURE 1:**
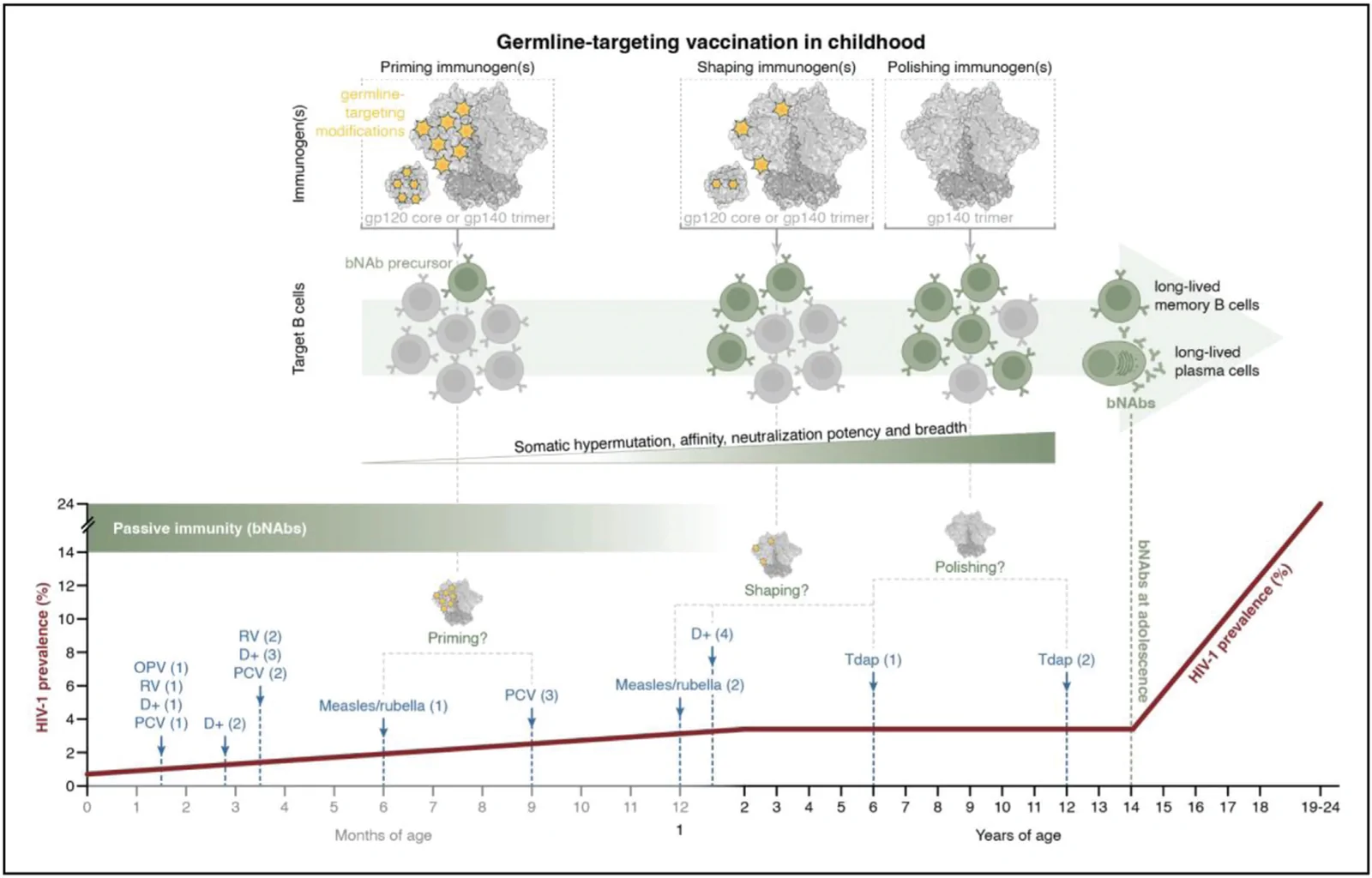
Germline-targeting, shaping, and polishing vaccination in early life. Priming immunogens with substantial epitope changes (indicated by stars) are followed by shaping immunogens with fewer changes and polishing immunogens that resemble native wild-type, virion-associated Env. The vaccination sequence is aimed at coaching B cells to eventually become long-lived bNAb-producing plasma cells. The schematic timeline of early life includes the prevalence of HIV-1 over time in a high-risk region. The early window for passive bNAb administration is shown, as is the sequential, active HIV-1 vaccination schedule outlined above. The different germline-targeting immunogens could be incorporated into the *Expanded Programme on Immunization* (EPI) schedule, that is, given at times when the children receive a scheduled, approved vaccine. The ones indicated on the timeline are: OPV, oral polio vaccine; RV, rotavirus vaccine; D+, diphtheria, tetanus, acellular pertussis, inactivated polio vaccine, *Haemophilus influenzae* type B and hepatitis B combined; PCV, pneumococcal conjugated vaccine; Tdap, tetanus, reduced strength diphtheria and acellular pertussis vaccine. The time points shown are based on the EPI South Africa revised childhood immunization schedule (January 2024).

An affordable vaccine delivered in early life that creates life-long immunity remains one of the best ways to counter this pandemic. To be protective against infection, a vaccine should be able to induce broadly neutralizing antibodies (bNAbs), that is, antibodies to one or preferably several epitopes that neutralize a substantial proportion of currently circulating HIV-1 strains [1,2]. There are major immunologic hurdles to the induction of bNAbs, and no vaccine evaluated so far in efficacy trials has been able to do so [1,2]. Nonetheless, there has been substantial progress in the design and evaluation of new, envelope glycoprotein (Env)-based immunogens that can recruit and activate naive B cells with the intrinsic capacity to become bNAb-secreting plasma cells. This strategy is termed ‘germline-targeting’ (Fig. 1). While it is by no means simple, this approach is currently the most advanced route towards the goal of creating a vaccine able to elicit bNAbs at titers that could protect children and adults from HIV-1 infection. Germline-targeting immunogens can be based either on Env fragments that optimally present selected epitopes or on stabilized, engineered versions of the Env trimer, which is the viral protein that mediates attachment and entry into CD4+ target cells and the sole target for bNAbs. Here, we review recent advances in the design and evaluation of the germline-targeting vaccine strategy. We address how such vaccines could be used in infants and adolescents to induce bNAbs prior to sexual debut.

## BROADLY NEUTRALIZING ANTIBODIES

The rationale for attempting to develop bNAb-inducing vaccines derives from studies of people with HIV-1 (PWH). Some untreated PWH (~20 to 50% in different cohort studies) naturally develop bNAbs over a multi-year period of co-evolution of the virus and the humoral immune system [3,4]. Thus, early in infection, the virus induces narrow-specificity NAbs, which drive the selection of escape variants that, in turn, push the antibody response towards neutralization breadth over multiple cycles of adaptation. The sequential rounds of affinity maturation and somatic hypermutation (SHM) eventually create mature B cells that secrete bNAbs [3,4]. Of note is that the process of bNAb development in serum occurs quicker and more frequently in children than adults, and sometimes as soon as 1–2 years after infection. It is this natural route of bNAb induction that the germline-targeting vaccine strategy seeks to mimic. The speedier sequence of events in children is one reason to evaluate such vaccines in that population.

The isolation and characterization of monoclonal bNAbs from PWH have identified multiple sites of vulnerability that, in aggregation, cover almost all of the exposed surface of the Env trimer [2,5]. These epitope clusters include: the binding site for CD4, the primary HIV-1 receptor; the trimer apex that incorporates components of the V2 domain and nearby glycans; the V3-N332 glycan patch on the outer domain; the gp120–gp41 interface, including the fusion peptide; and the membrane proximal external domain (MPER).

Characterizing these natural bNAbs has shown why it is so difficult to induce them by vaccination. This topic has been reviewed extensively elsewhere [2]. In summary, the main obstacles to bNAb induction include: viral sequence and hence antigenic diversity; the epitope-shielding effect of glycans; and the instability of the Env trimer. Accordingly, most HIV-1 bNAbs have unusual properties compared to most antibodies. For example, V2-apex-directed bNAbs typically need very long CDRH3 loops to penetrate the glycan shield and reach underlying, antigenic protein surfaces. In contrast, CD4bs-directed bNAbs of the VRC01-class have uncharacteristically short CDRL3 loops so as to avoid clashes with the shielding N276-glycan. Unusual insertion and/or deletion events in the CDRH1, CDRL1, and FWR3 elements of antibodies are additional devices to minimize clashes with glycans and/or strengthen interactions with the epitope surface. Furthermore, many bNAbs have polyreactivity or autoreactivity properties, which may limit their induction via counter-selection during B-cell ontogeny. Overall, naive B cells carrying bNAb precursors as their B-cell receptor (BCR) are only rarely present in the naive human B-cell repertoire [2]. Vaccine design must therefore be based on an understanding of the obstacles to bNAb induction that inform hypotheses on how to overcome them.

## BROADLY NEUTRALIZING ANTIBODY DEVELOPMENT IN INFANTS

While direct comparisons with adult cohorts are difficult due to the different modes and timing of virus acquisition involved, it is generally accepted that bNAb development is relatively rapid and frequent in HIV-1-infected infants, based on studies in multiple pediatric cohorts [6–10]. The plasma bNAbs present in young children are also more often directed against multiple epitopes, whereas in adults, typically only a single site is targeted.

It is not yet clear whether the characteristics of the developing immune system account for such differences, or whether the properties of vertically transmitted viruses also or instead play a key role here. The potential immunologic advantages of the early life immune system for bNAb development include, but may not be limited to, greater adenosine deaminase activity; a higher number of variable gene region targets; a greater frequency of naive B cells; Th2-skewed cellular immunity; and distinct modes of antigen presentation [11–15].

Relevant virologic factors may include favorable Env sequences and/or antigenic structures that are selected for during vertical, as opposed to horizontal, transmission, and/or that evolve during cycles of escape from maternal humoral immunity [16,17]. As an aside, but of possible relevance, is that the HIV-1 Env sequence used as the basis of the germline-targeting GT1.1 trimer was isolated from an infant: the BG505 clade A virus [8,18–20]. The GT1.1 immunogen has shown promise in preclinical and clinical studies (see below; [21,22^▪^]).

The many bNAbs isolated from adults generally share common characteristics, including high SHM rates, frequent insertions and/or deletions, long CDRH3 regions, and autoreactivity or polyreactivity (see above; [2,5]). The only two monoclonal bNAbs derived from children are somewhat different, however. The V3 glycan-specific bNAb BF520 isolated from an infant 1 year after infection is unusual in that it underwent only limited SHM and has no insertions or deletions [23]. Furthermore, the mutations required for BF520 neutralization breadth are considered improbable and are located outside the CDRH3 region that is the site of breadth-associated evolutionary change in adult bNAbs [24]. Hence, there may be distinctive pathways of bNAb development in children, although more examples would be needed to firm up this supposition. Support does come from a second anti-V3 glycan-specific bNAb, AIIMS-P01 isolated from a 9-year-old child. This antibody has remarkable neutralization breadth despite a low degree of SHM [25,26]. Clearly, obtaining additional monoclonal bNAbs from children that target other epitopes would increase our knowledge in this area.

Despite the acknowledged limitations to our understanding of HIV-1 infection during early life, the potentially lower immunologic barriers to the development of bNAb precursor lineages within the developing immune system does support pediatric immunization strategies. The concept here is that the successful priming of rare bNAb lineages in early life, followed by appropriate boosting during the period of routine childhood vaccine administration, may lead to protective plasma bNAb titers and/or immune memory before sexual debut.

## PASSIVE IMMUNIZATION WITH BROADLY NEUTRALIZING ANTIBODIES

HIV-1 bNAbs serve as critical templates for vaccine design (see below). However, passively administered monoclonal bNAbs may also play a role in prophylactic, therapeutic, or cure strategies [27]. In the pediatric setting, a paradigm is provided by Nirsevimab (Beyfortus, D25; [28–30]), an engineered, neutralizing monoclonal antibody (MAb) used to prevent respiratory syncytial virus (RSV) disease in infants. Multiple HIV-1 bNAbs are currently being evaluated prophylactically, alone and in combination.

The epidemiology of pediatric HIV-1 acquisition risk is bimodal. Perinatal transmission risk begins at birth, usually ends when the infant is weaned from breastfeeding, but then increases steeply during adolescence upon sexual debut. The time-limited window of acquisition risk for infants between birth and weaning provides an opportunity to use existing bNAbs, likely in combination, for passive immunization to protect against breastmilk transmission. The chosen bNAbs typically have Fc region modifications to increase their longevity within the body after intramuscular delivery. That approach has been successful for passive protection of human infants by anti-RSV MAbs [29,30], and of nonhuman primates by HIV-1 bNAbs [31–33]. The AMP trial showed that the anti-CD4 binding site bNAb VRC01 could protect adults against infection, when present at high enough concentrations and when the infecting virus was sensitive to it [34]. However, another conclusion from this trial was that bNAb combinations would be required to overcome pre-existing antibody resistance in the currently circulating population of HIV-1 variants [35]. Thus, a bNAb double or even triple combination would provide greater neutralization breadth and higher resistance barriers, but would still be cost-effective for both exposed infants [36] and those born in high HIV-1 prevalence settings [37]. Additional pediatric clinical trials to evaluate the value of this concept are needed, with the goal of identifying a product suitable for licensure and application.

Long-duration bNAbs could also be used for passive immunization concurrently with a germline-targeting vaccine during the childhood vaccination schedule. The concept here is to provide passive bNAb protection against breastmilk transmission, while vaccination-driven active immunity could develop in time to be protective during adolescence (Fig. 1). Antibody interference with vaccine uptake by the formation of immune complexes could be a concern for this strategy; while an initial experiment suggested otherwise, more studies using newer bNAbs and vaccine reagents are needed [38].

## GERMLINE VACCINATION STRATEGIES

How can bNAbs be induced through active vaccination? The vaccines studied in efficacy trials failed to elicit narrow-specificity NAbs against primary HIV-1 strains, let alone bNAbs. A breakthrough came from studies of how Env interacts with the inferred germline precursors of bNAbs, that is, the putative BCR on the naive precursor B cells from which the bNAb-producing mature B cells originate. A key observation made more than a decade ago was that the then-existing Env protein designs used in clinical trials could not bind detectably to bNAb precursors [39–45]. The resulting hypothesis was that Env proteins needed to be specifically modified to bind bNAb precursors – what we now refer to as ‘germline-targeting’ (Fig. 1).

The initial germline target was the VRC01-class of B cells and bNAbs, which use a VH1–2-derived heavy chain coupled to a short, 5-amino-acid CDRL3 in the light chain. Three such immunogens have progressed to phase 1 trials. The first, eOD-GT8, is a version of the outer domain (OD) of gp120, modified specifically to facilitate high-affinity binding to VRC01-class precursors. Data from pre-clinical models and human trials have supported the use of this design, both as an adjuvanted protein and when delivered as an mRNA construct [42,46,47^▪^]. The second such immunogen, 426c.Mod.Core, is a monomeric gp120 core also modified to increase its interaction with VRC01-class precursors [43]; clinical studies are underway to evaluate its utility in humans (HVTN301; NCT05471076). Finally, the GT1.1 protein is based on the stabilized BG505 SOSIP trimer [18], again engineered to enhance its interaction with VRC01-class precursors [21,48]. This trimer has shown promise in preclinical and human studies [21,22^▪^]. Thus, in the IAVI C101 phase 1 human trial, GT1.1 induced VRC01-class memory B cells efficiently and triggered substantial on-path affinity maturation [22^▪^].

Although it is now established that CD4bs-directed bNAb precursors can be primed, the next challenge is to ‘shape and polish’ these responses into maturity; bNAb-producing B cells are the much-desired end product. Shaping and polishing involve exposing B cells to Env molecules that more closely resemble wild-type trimers; their modifications include reverting the germline-targeting mutations to wild-type residues and restoring glycans that were removed to enhance access to germline bNAb epitopes. The shaping and polishing steps should also involve exposing the targeted B cells to sequence diversity that reflects circulating strains. The single biggest obstacle that must be overcome for VRC01-class bNAbs is the N276 glycan, which is absent, by design, from all VRC01-class priming immunogens tested to date. The shaping and polishing immunogens must, therefore, be designed to achieve the goal of accommodating this glycan. Initial shaping steps have been successful in preclinical studies of eOD-GT8 [49,50], 426c.Mod.Core [51], and GT1.1 [21,52].

Clinical trials are underway to evaluate candidate shaping and/or polishing immunogens for each of the above three VRC01-class priming designs (Table 1). In the IAVI G002 trial, a first-boost immunogen, core-g28v2, further matured VRC01-class B cells that had been primed by eOD-GT8 [47^▪^]. The HVTN301 and HVTN320 trials explore two different shaping strategies following 426c.Mod.Core priming, one involving another gp120 core protein, HxB2.WT.Core (HVTN320; NCT06796686), the other using the GT1.1 trimer as a first shaping immunogen (HVTN301; NCT05471076). Finally, in the IAVI C110 and C107 trials (NCT05863585 and NCT05983874), individuals who received a (BG505) GT1.1 protein prime were then given two doses of the fully glycosylated BG505 SOSIP.664 wild-type trimer. The goal was to gauge whether VRC01-class B cells could be matured, even without the use of a specifically designed shaping immunogen.

**Table 1:** Completed and ongoing clinical trials with VRC01-class targeting priming immunogens

Trial name	NCT identifier	Immunogen(s)	Delivery platform	Population	Location	Result	Ref
IAVI G001	NCT03547245	eOD-GT8 60mer	Protein + AS01b	Healthy adults	USA	Vaccine-induced VRC01-class B cells	[46]
IAVI G002	NCT05001373	eOD-GT8 60mer Core-g28v2 60mer	mRNA-LNPs	Healthy adults	USA	Vaccine-induced and matured VRC01-class B cells	[47^▪^]
IAVI G003	NCT05414786	eOD-GT8 60mer	mRNA-LNPs	Healthy adults	Rwanda, South Africa	Vaccine-induced VRC01-class B cells	[47^▪^]
IAVI G004	NCT06694753	eOD-GT8 60mer Core-g28v2 60mer	mRNA-LNPs	Healthy adults	South Africa	n.p.	n.p.
HVTN301	NCT05471076	426c.Mod.Core-C4b BG505 GT1.1	Protein + 3M-052/Alum	Healthy adults	USA	n.p.	n.p.
HVTN316	NCT06613789	426c.Mod.Core-C4b	Protein + 3M-052/Alum	Infants	South Africa	n.p.	n.p.
HVTN320	NCT06796686	426c.Mod.Core-C4b HxB2.WT.Core-C4b	Protein + 3M-052/Alum	Healthy adults	USA	n.p.	n.p.
HVTN807	NCT06006546	426c.Mod.Core-C4b	Protein + 3M-052/Alum	Adult PWH	USA	n.p.	n.p.
IAVI C101	NCT04224701	BG505 GT1.1	Protein + AS01b	Healthy adults	USA, Netherlands	Vaccine-induced and matured VRC01-class B cells	[22^▪^]
IAVI C107 IAVI C110	NCT05863585 NCT05983874	BG505 GT1.1 BG505 SOSIP.664	Protein + AS01b Protein + 3M-052/Alum	Healthy adults	USA, Netherlands	n.p.	n.p.
RENEW-SHCS	n.a	BG505 GT1.1	Protein + 3M-052/Alum	Adult PWH	Switzerland	n.p.	n.p.
RENEW-CAP	n.a	BG505 GT1.1	Protein + 3M-052/Alum	Adult PWH	South Africa	n.p.	n.p.
BRILLIANT-011	n.a	BG505 GT1.1 426c.Mod.Core-C4b	Protein + SMNP	Healthy adults	South Africa	n.p.	n.p.

In some trials, the priming stage is followed by a shaping or polishing immunogen. n.a., NCT identifier not available; n.p., not published; PWH, people with HIV-1.

With proof-of-concept for germline-targeting for VRC01-class B cells now established, and efforts for shaping and polishing well under way, attention has turned towards other epitopes. The goal here is to create a germline-targeting vaccine(s) for multiple bNAb epitopes. Various strategies to do this are reviewed elsewhere [53].

## GERMLINE-TARGETING IMMUNIZATION IN EARLY LIFE

The potential advantages of a more naive B-cell repertoire, distinct SHM patterns, and the more frequent and rapid emergence of bNAbs post-infection, when paired with the 10+ years of limited or absent exposure to HIV-1, make early childhood a promising age group for germline-targeting vaccines. In a proof-of-concept study, priming with the BG505 GT1.1 SOSIP trimer followed by wild-type BG505 SOSIP.664 boosting induced VRC01-like bNAb precursors in infant nonhuman primates [54^▪^]. The infant animal data mirrored and perhaps improved on what was seen in adult nonhuman primate and human trials of the same or similar immunogens [54^▪^].

A strong practical consideration applies to adding a sequential germline-targeting, shaping, and polishing HIV-1 vaccine regimen into the multidose childhood vaccination programs used worldwide (Fig. 1). By doing so, existing infrastructures and routine health visits in areas of the world most impacted by HIV-1 could be leveraged [55]. Moreover, as adolescence is the highest risk period for HIV-1 acquisition during the human lifespan, the successful use of an effective vaccine during childhood would have a major impact on the course of the pandemic. Nonetheless, there are limitations, and there will be challenges. Thus, there are as yet no data on the precursor frequency of, for example, VRC01-class germline precursors in infants. And it is by no means certain that the sequential germline-targeting immunogens could, in practice, be given simultaneously with approved vaccines in, for example, the EPI schedule. Even so, we argue that there is a strong justification for the continued inclusion of infants and children in trials of the present and future germline-targeting HIV-1 vaccine regimens. At least one such trial is now being planned (NCT06613789; Table 1).

## CONCLUSION

The Dutch proverb ‘*jong geleerd is oud gedaan*’ (literal translation: ‘learnt young is done old’) tells us that what we do early in life can have long-term benefits as we age. That wisdom can apply to germline-targeted vaccination against HIV-1. The bNAb precursor B cells present in infants might be particularly susceptible to strategies intended to mature them into bNAb-producing B cells during the long window available before the high risk posed by sexual debut during adolescence. The accumulating data on how the immune system develops therefore support the further evaluation of germline-targeting vaccination in infants. Moreover, the general concept of vaccination in early life to protect the child, adolescent, and adult against life-altering infections is well established.

## Acknowledgements

*The authors’ research in this area is supported by NIH grants P01 AI117915 (to S.R.P.) and P01 AI197052 (to J.P.M), and by grants from the Gates Foundation: INV-008818, INV-048573, INV-083908, INV-063951 and INV-091003 (to R.W.S. and J.P.M.).*

### Financial support and sponsorship

*None.*

### Conflicts of interest

*S.R.P. serves as a consultant to Pfizer, Merck, Moderna, and Kamada and leads sponsored programs with Pfizer and Kamada regarding cytomegalovirus vaccines and prevention. R.W.S. and J.P.M. are listed as inventors on patents related to HIV-1 immunogens.*

## REFERENCES AND RECOMMENDED READING

Papers of particular interest, published within the annual period of review, have been highlighted as:

▪ of special interest
▪▪ of outstanding interest
